# Imaging manifestations of juvenile hyaline fibromatosis: a case report and literature review

**DOI:** 10.1259/bjrcr.20210167

**Published:** 2022-01-07

**Authors:** Jinfen Yu, Linsheng Wang, Jing Tian, Xuewen Yu, Lixin Sun

**Affiliations:** 1 Shandong Second Provincial General Hospital, Jinan, Shandong, PR China

## Abstract

**Objective:**

Juvenile hyaline fibromatosis (JHF) is an autosomal recessive condition caused by a mutation in capillary morphogenesis gene 2 (CMG2) on chromosome 4q21. JHF is an extremely rare genetic disorder, and fewer than a hundred cases have been reported worldwide. In this case report, the clinical features, histopathological features and imaging manifestations of a case of JHF are presented. We present imaging manifestations of one case of JHF to deepen the radiologist’s understanding of this condition. The histopathological feature of JHF is hyaline degeneration involving skeletal muscle. Therefore, the lesion has a slightly high density on CT imaging, iso- or hypointense signal on *T*_1_WI and hypointense signal on *T*_2_WI. The boundary between the lesion and skeletal muscle is unclear.

**Methods:**

An 8-year-old male (Case 1) was examined in our department with a complaint of multiple masses on the head, neck and back in 2021. The boy was the only child of his parents and was delivered at 40 weeks gestation by caesarean section. His parents were non-consanguineous.

**Results:**

JHF displays multiple slowly or rapidly growing subcutaneous nodules. The imaging manifestations can reflect histopathological components, including nodular connective tissue and amorphous, partially calcified hyaline material.

## Introduction

Juvenile hyaline fibromatosis (JHF) is an autosomal recessive condition caused by a mutation in capillary morphogenesis gene 2 (CMG2) on chromosome 4q21.^1,2^ JHF is an extremely rare genetic disorder, and fewer than a hundred cases have been reported worldwide.^3^ JHF is characterised clinically by multiple papules, nodules or masses in the head and back; hypertrophy of the gingiva; and flexural contractures of the large joints.^4,5^ JHF is an early onset disease that typically appears in the first 4 years of life and often shows its first signs early, even at birth.^4,6^ The diagnosis is confirmed by demonstration of hyaline deposition in the lesion.^7,8^

From the first report of JHF in 1873 to relevant literature reports in 2020, all of the studies focused on histopathology, gene detection, immunohistochemical analysis, clinical treatment and follow up of JHF, whereas imaging manifestations of JHF were rarely reported.^3–5,7–9^ We present imaging manifestations of a case of JHF to deepen the radiologist’s understanding of this condition. The literature on JHF and infantile systemic hyalinosis is also reviewed.

## Case report

An 8-year-old boy with multiple masses on the head, neck and back since birth was brought to the ENT Department of Shandong Second Provincial General Hospital.

**Figure 1.**
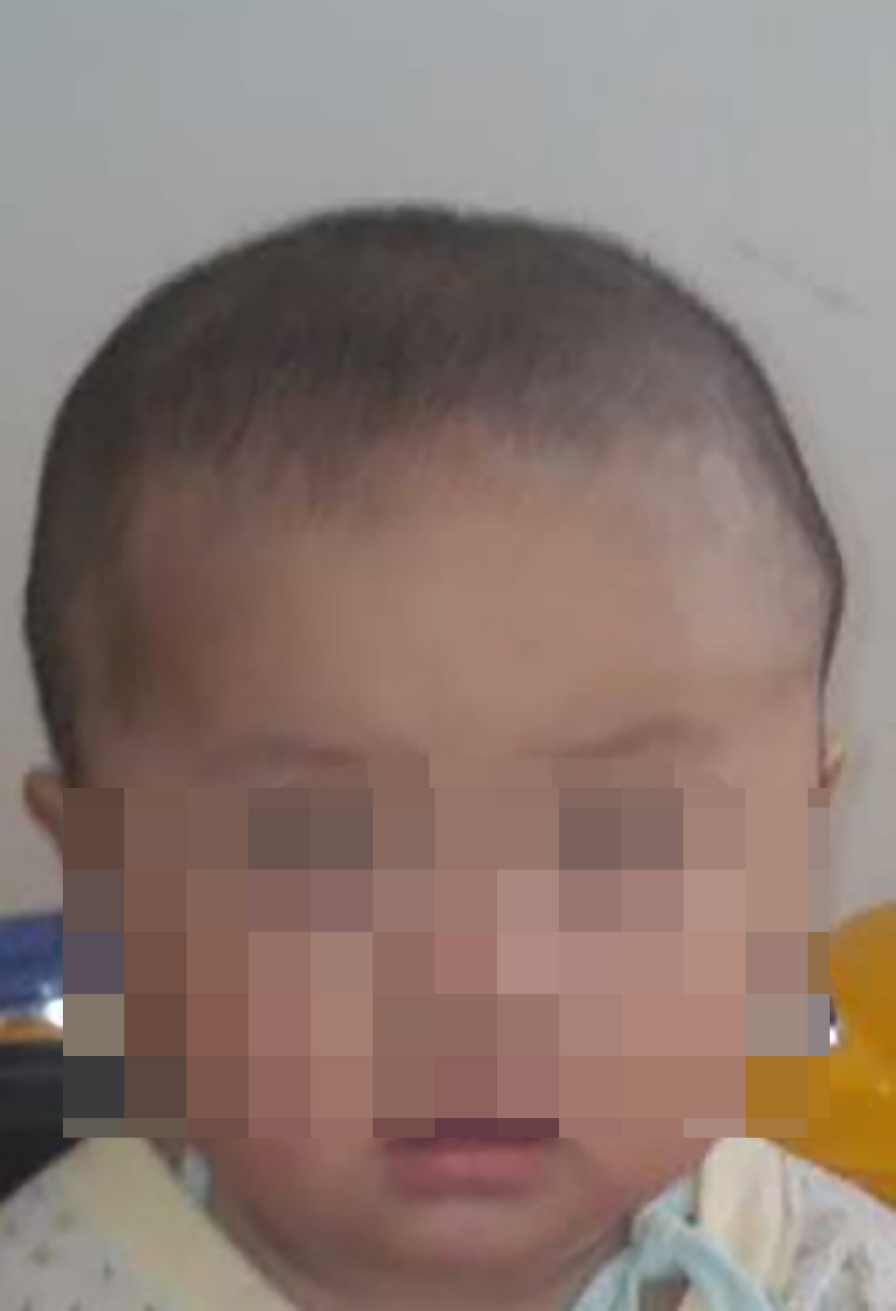
Left temporal scalp nodules.

The boy was the only child for his parents and was delivered at 40 weeks gestation by caesarean section. His parents were non-consanguineous. His birth weight was 3.5 kg, and he was 50 cm long. Multiple nodules were present on his head at birth. His parents found multiple nodules on his head, neck and back when he was 2 or 3 months old. All the nodules became larger but grew very slowly. In addition, the nodules have remained painless to date. The boy is very clever, and he gets top marks on his exams. Unfortunately, pre-operative photos of the patient were not available due to various reasons, and only one photo of the patient at the age of 1 was available (Figure 1).

### Examination

The boy was referred for head, neck and chest CT and MRI examination, which revealed multiple masses on the head, neck and back, and the bone adjacent to the lesion was hyperplastic. The scan ranged from the crown of the head to the upper abdomen and included all lesions.

**Figure 2.**
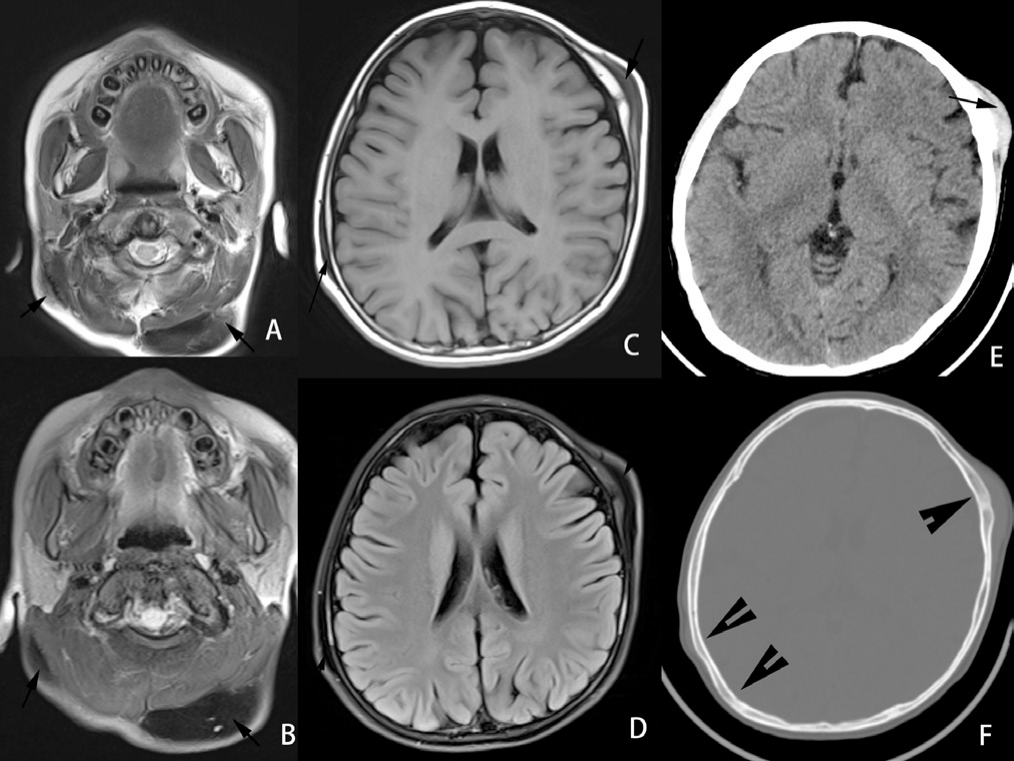
An 8-year-old male with juvenile hyaline fibromatosis. CT and MR imaging showed multiple subcutaneous lesions on the scalp (**A–F**). The left temporal and right temporoparietal skull bone had hyperplasia and sclerosis (**F**). The lesions showed iso- or hypointense signals on *T*_1_WI and hypointense signals on *T*_2_WI (**A–D**). The masses showed slightly high density on CT (**E**). *T*_1_WI, *T*_1_ weighted imaging; *T*_2_WI; *T*_2_ weighted imaging,

**Figure 3.**
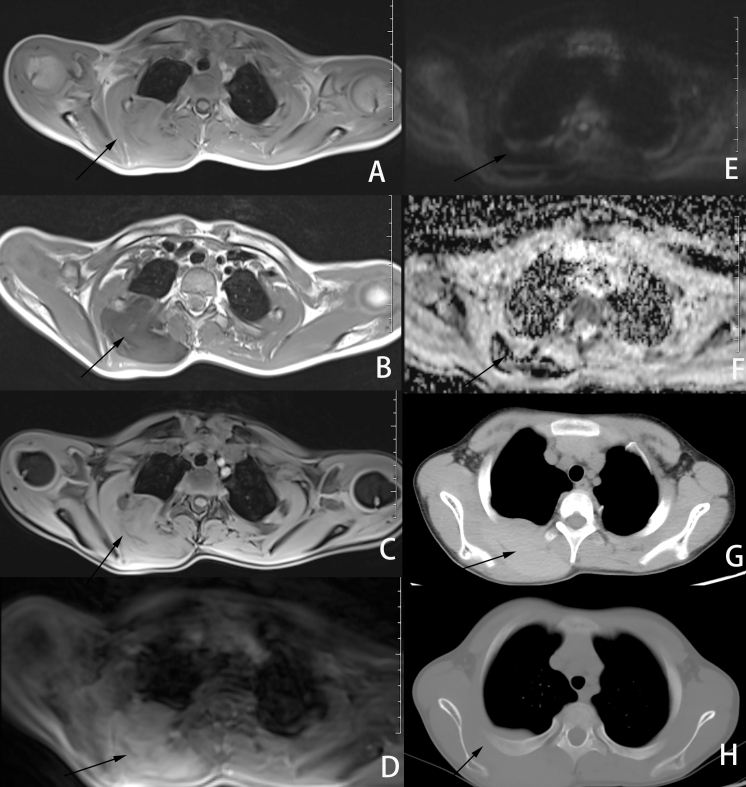
The soft tissue lesion on the right back involved intercostal muscles (**A–G**). The adjacent rib was thickened (**F**). No obvious signal change was noted after fat pressing on *T*_1_WI (**C**). The lesion showed hypointense signal on *T*_1_WI and hypointense signal on *T*_2_WI (**A–B**). The lesions showed hyperintensity on FS-*T*_1_WI after the contrast agent was administered (**D**). The masses show hypointense signals on DWI and ADC maps (**E–F**). The mass lesion showed slightly high density on CT (**G**). ADC, apparent diffusion coefficient; DWI, diffusion-weighted imaging; FS, fat saturated; *T*_1_WI, *T*_1_ weighted imaging; *T*_2_WI; *T*_2_ weighted imaging.

**Figure 4.**
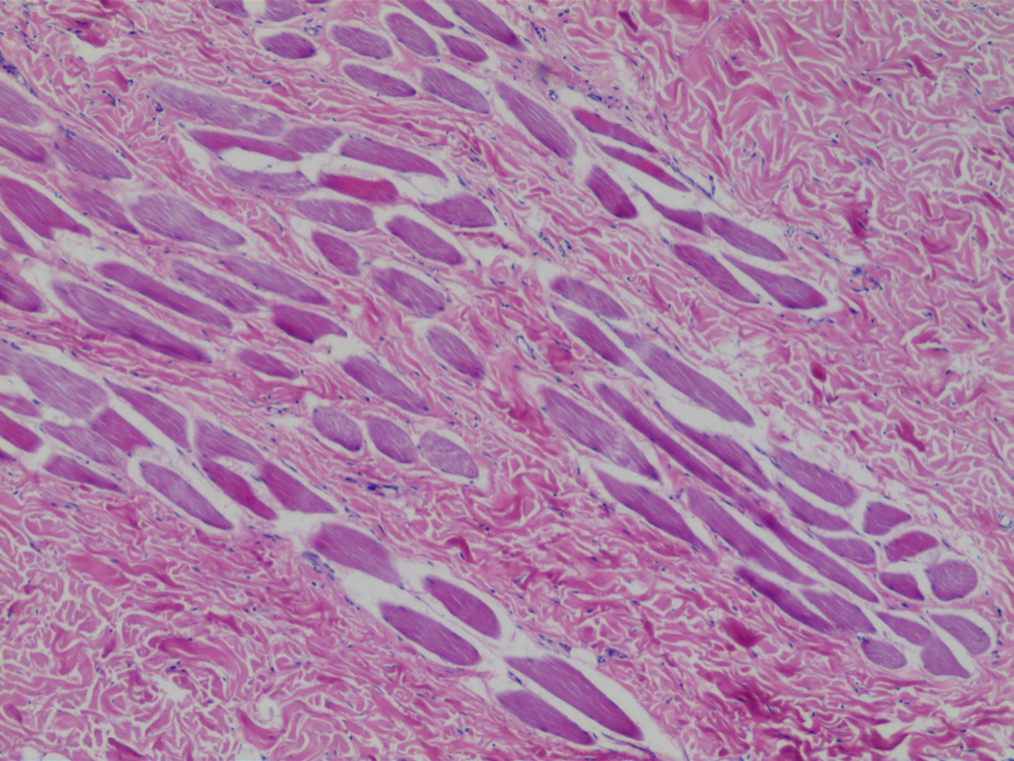
High-power histopathological imaging showed hyaline deposition in the lesion, and the lesion invaded the surrounding skeletal muscle with an ill-defined boundary (haematoxylin and eosin, ×20).

CT and MRI imaging showed multiple soft tissue masses in the parietal, left frontal and occipital aera, left maxillofacial region, periauricular region, subcutaneous fatty space at the level of the second to fourth cervical vertebral body, fatty space behind C3 to C5 of the cervical spinous process, right back muscle and intercostal muscles. The masses were accompanied by hyperosteosclerosis of the adjacent skull or rib. The masses showed iso- or hypointense signals on *T*_1_WI and hypointense signals on *T*_2_WI. The masses showed hypointense signal on DWI and the ADC map. The mass gradually strengthened with the prolongation of time after the contrast agent was administered. All masses showed mild hyperdensity with hyperosteosclerosis of adjacent bone on CT (Figures 2 and 3). All lesions were indistinguishable from adjacent muscles, which was consistent with the pathological features. High-power histopathological imaging showed hyaline deposition in the lesion, and it invaded the surrounding skeletal muscle with an ill-defined boundary (Figure 4). No abnormalities were observed in the epigastrium, which was confirmed with abdominal ultrasound. No abnormalities were observed by pelvic cavity ultrasound.

## Discussion

JHF is a rare hereditary disorder first reported as “mollusum fibrosum” in 1873 by Murray.^9^ The name “juvenile hyaline fibromatosis” was first introduced by Drescher et al. in 1967.^10^ Drescher et al officially named the condition JHF in 1969.^11^ JHF is a relatively mild presentation of hyaline fibromatosis syndrome, which is a connective tissue disease with two different clinical manifestations of similar pathophysiology: infantile hyaline fibromatosis (IHF) and JHF. IHF with involvement of the viscera is the most lethal form.^2,12–16^ Patients with juvenile hyaluronidosis can survive into adulthood, whereas patients with IHF suffer from intractable diarrhoea and recurrent infections leading to early death.^16^ Fortunately, our patient’s viscera were not affected by the lesion.

The clinical features of JHF include gingival hyperplasia, osteopaenia, osteolytic bone lesions, papular and nodular skin lesions, and joint contractures.^4,5,16,17^ Skin lesions are present at birth or develop in early childhood. The diagnosis is confirmed by demonstration of hyaline deposition in the lesion.^7,8^ In our case, high-power histopathological imaging revealed hyaline deposition in the lesion, and the lesion invaded the surrounding skeletal muscle with an ill-defined boundary. Therefore, lesions were indistinguishable from adjacent muscles on CT and MRI imaging. Gene detection reveals a mutation in capillary morphogenesis gene 2 (CMG2) on chromosome 4q21. CMG2 encodes a protein involved in basement membrane matrix assembly, in particular collagen type VI homeostasis, and endothelial cell morphogenesis.^1,2,16,18^ Our patient had multiple masses on the head, neck and back, which was a common finding in this condition. The masses were accompanied by hyperosteosclerosis adjacent to the skull or rib. The masses showed iso- or hypointense signals on *T*_1_WI and hypointense signals on *T*_2_WI. The mass gradually strengthened with time after the contrast agent was administered. All lesions showed slightly high density on CT. We assume that these imaging manifestations are closely related to histopathological findings, namely, nodular connective tissue and amorphous, partially calcified hyaline material.^16,19^

Generally, gingival hyperplasia is observed in JHF, and cases without gingival hyperplasia have only rarely been reported.^20^ However, no gingival hyperplasia was noted in our case.

JHF displays multiple slowly or rapidly growing subcutaneous nodules.^16,20^ Our patient presented with a painless swelling mass.

The treatment of JHF is generally considered to be unsatisfactory. Recurrence may occur after excision of the mass.^6,16^ Gingivectomy is useful for gingival hyperplasia. Frequent periodontal visits are important for maintaining oral hygiene and decreasing the growth rate of gingiva in JHF.^19^

## Learning points

First, JHF is characterised clinically by multiple papules, nodules or masses in the head and back; hypertrophy of the gingiva; and flexural contractures of the large joints at birth or in the first 4 years of life.

Second, the lesion involves skeletal muscle with an ill-defined boundary. The lesion may or may not involve viscera and gingiva.

Finally, hyaline deposition in the lesion results in its characteristic radiographic appearance.
